# Antibiotic stewardship in the emergency department setting: Focus on oral antibiotic selection for adults with skin and soft tissue infections

**DOI:** 10.1093/ajhp/zxae163

**Published:** 2024-07-04

**Authors:** Heather M Draper, Michael J Rybak, Kerry L LaPlante, Thomas Lodise, George Sakoulas, Muriel Burk, Francesca E Cunningham

**Affiliations:** Trinity Health Grand Rapids, Grand Rapids, MI, USA; Anti-Infective Research Laboratory, College of Pharmacy and Health Sciences, Wayne State University, Detroit, MI; School of Medicine, Wayne State University, Detroit, MI, USA; Department of Pharmacy Practice, University of Rhode Island, Kingston, RI; Rhode Island Infectious Diseases Research Program, Providence Veterans Affairs Medical Center, Providence, RI, USA; Department of Pharmacy Practice, Albany College of Pharmacy and Health Sciences, Albany, NY, USA; Sharp Rees Stealy Medical Group, San Diego, CA; Department of Pediatrics, UCSD School of Medicine, La Jolla, CA, USA; Department of Veterans Affairs, Hines, IL, USA; Department of Veterans Affairs, Hines, IL, USA

**Keywords:** antimicrobial stewardship, emergency department, medication-use evaluation, pharmacist, skin and soft tissue infections

## Abstract

**Purpose:**

An advisory panel of experts was convened by the ASHP Foundation as a part of its Medication-Use Evaluation Resources initiative to provide commentary on an approach to antibiotic stewardship in the treatment of skin and soft tissue infections (SSTIs), with a focus on oral antibiotics in the emergency department (ED) setting for patients who will be treated as outpatients. Considerations include a need to update existing guidelines to reflect new antibiotics and susceptibility patterns, patient-specific criteria impacting antibiotic selection, and logistics unique to the ED setting.

**Summary:**

While national guidelines serve as the gold standard on which to base SSTI treatment decisions, our advisory panel stressed that institutional guidelines must be regularly updated and grounded in local antimicrobial resistance patterns, patient-specific factors, and logistical considerations. Convening a team of experts locally to establish institution-specific guidelines as part of a comprehensive antibiotic stewardship program can ensure patients receive the most appropriate oral therapy for the outpatient treatment of SSTIs in patients visiting the ED.

**Conclusion:**

SSTI treatment considerations for antibiotic selection in the ED supported by current, evidence-based guidelines, including guidance on optimal oral antibiotic selection for patients discharged for outpatient treatment, are a useful tool to improve the quality and efficiency of care, enhance patient-centric outcomes and satisfaction, decrease healthcare costs, and reduce overuse of antibiotics.

**Key Points**

- The emergency department (ED) poses unique challenges for antibiotic stewardship in the treatment of SSTIs in patients who are candidates for outpatient oral therapy, including the need to treat empirically, lack of patient follow-up, and medication access issues.
- Institution-specific guidelines for SSTIs should be updated regularly to include new antibiotics, local antimicrobial resistance patterns, patient-specific clinical considerations, and logistics issues.
- Medication-use evaluation in the ED setting as part of a pharmacist-led antibiotic stewardship program can ensure effective guideline implementation.

Skin and soft tissue infections (SSTIs) are among the top 10 reasons patients present to the emergency department (ED).^1,2^ Among the individuals at elevated risk for SSTIs are those who are immunocompromised or have chronic conditions such as diabetes or lymphedema, and those with multiple comorbidities.^3^ SSTIs include both purulent infections (eg, abscesses and other chronic wounds) and nonpurulent infections (eg, cellulitis and erysipelas).^3^ Acute bacterial skin and skin structure infections (ABSSSIs) are a specific subset of SSTIs designated by the US Food and Drug Administration to guide new antibiotic approvals and include major cutaneous abscesses (with a minimum lesion surface area of 75 cm^2^ and including edema, erythema, and induration), wound infections, and erysipelas.^4^ SSTIs pose substantial clinical challenges; inadequate or delayed treatment can lead to severe complications and contribute significantly to the healthcare system’s financial burden.^1^

In the ED, patient assessment decisions are largely empiric and made without guidance from current culture and susceptibility data. With the goal of optimal resolution and avoidance of return visits to the ED and/or hospitalization, empiric antibiotic selection is often initiated considering local resistance patterns, prior culture results, purulent versus nonpurulent characteristics, host innate immunity, drug-drug interactions, and prior antibiotic exposure in the patient. Treatment decisions should be guided by antibiotic stewardship principles, including treatment that is not overly broad and is directed at the most likely pathogens for the shortest treatment duration possible. Clinicians largely rely on expert guidelines to inform empiric antibiotic decisions for patients with SSTIs. Unfortunately, the Infectious Diseases Society of America (IDSA) SSTI treatment guidelines, considered the gold standard by clinicians, were last updated in 2014.^5^ As such, the guidelines are outdated concerning (1) newer antibiotics that have been approved and made available since that time (eg, dalbavancin, oritavancin, delafloxacin, tedizolid, and omadacycline); (2) changes in the epidemiology of methicillin-resistant *Staphylococcus aureus* (MRSA) in the community; (3) uptake of severity scoring classifications (eg, Eron/CREST and Dundee classifications) for patients presenting with cellulitis; (4) increased availability of rapid testing (eg, polymerase chain reaction for MRSA colonization screening); and (5) changes in resistance patterns among beta-hemolytic streptococci (eg, increased macrolide and clindamycin resistance).^6-8^

In addition to the lack of updated guidelines, the ED setting poses specific and unique logistical challenges for the care of patients presenting with SSTIs, such as (1) throughput considerations to optimize and enhance patient flow and satisfaction; (2) decisions on subsequent site of care (eg, hospital, observation unit, or outpatient setting); (3) prescription coverage of prescribed antibiotics for patients discharged to home immediately from the ED or after an abbreviated stay in the observation unit; (4) loss of a patient to follow-up; (5) inability to assess outcomes due to lack of electronic health record data exchange and interoperability across care settings and providers; and (6) reimbursement and site-of-care options for implementing higher-cost but more efficient treatment strategies (eg, ability to administer long-acting intravenous agents like dalbavancin or oritavancin in the ED as opposed to an infusion center). These site-of-care challenges are often exacerbated when healthcare finance analyses are siloed within service lines. These challenges and related considerations may directly affect the ED provider’s ability to transition the patient to the optimal treatment setting (eg, admission or discharge to home) in a timely manner and to minimize patient return to the ED or hospital due to lack of adherence, infection relapse, recurrence, or an adverse drug reaction.

In order to address these challenges and shortcomings in the current (ie, 2014) IDSA SSTI guidelines in terms of antibiotic stewardship and updated treatment standards, an advisory panel (AP) of experts was convened by the ASHP Research and Education Foundation as a part of its Medication-Use Evaluation Resources initiative to review and suggest an approach to evidence-based treatment of SSTI in the ED and to identify key considerations when conducting a medication-use evaluation for SSTI treatment in the ED that supports quality improvement efforts to achieve optimal outcomes (Box 1). The AP comprised infectious diseases and emergency medicine pharmacy specialists, infectious diseases academicians and researchers, drug use and safety pharmacists, and an infectious diseases physician. During that process, it became evident that it was critical to incorporate antibiotic stewardship principles when implementing guidelines locally and recognize challenges unique to the setting. This commentary provides a summary of the AP consensus opinion and highlights potential antibiotic stewardship–directed initiatives in the ED setting, with a focus on oral antibiotic selection for adults with SSTIs.

**Box 1.** Advisory Panel

**Muriel Burk, PharmD** Clinical Specialist Pharmacist, Program Manager Department of Veterans Affairs Hines, IL **Francesca E. Cunningham, PharmD** Director, Center for Medication Safety Associate Chief Consultant, PBM Department of Veterans Affairs Hines, IL **Heather M. Draper, PharmD, BCPS, BCEMP** Clinical Pharmacy Specialist, Emergency Medicine Trinity Health Grand Rapids Grand Rapids, MI **Toni Fera, BSPharm, PharmD** Consultant Pittsburgh, PA **Kerry L. LaPlante, PharmD., FCCP, FIDSA, FIDP** Dean and Professor, University of Rhode Island College of Pharmacy Infectious Diseases Pharmacotherapy Specialist Director, Rhode Island Infectious Diseases Research Program, Providence Veterans Affairs Medical Center Adjunct Professor of Medicine, Brown University Kingston and Providence, RI	**Thomas Lodise, PharmD, PhD** Professor, Albany College of Pharmacy and Health Sciences Infectious Diseases Clinical Pharmacy Specialist, Stratton VA Medical Center Albany, NY **Barbara B. Nussbaum, BSPharm, PhD** ASHP Foundation Bethesda, MD **Michael J. Rybak, PharmD, MPH, PhD** Professor of Pharmacy and Director, Anti-Infective Research Laboratory, Department of Pharmacy Practice Wayne State Eugene Applebaum College of Pharmacy and Health Sciences Adjunct Professor of Medicine, Division of Infectious Diseases, Center for Emerging and Infectious Diseases, School of Medicine, Wayne State University Detroit, MI **George Sakoulas, MD** Infectious Disease, Sharp Rees Stealy Medical Group San Diego, CA Department of Pediatrics, UCSD School of Medicine La Jolla, CA

## Updating guidance for treatment of SSTIs in ED setting

**Figure 1.**
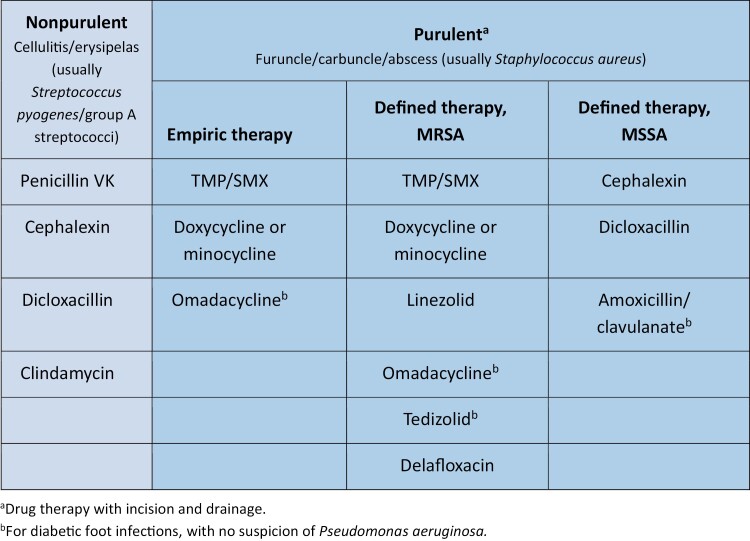
Oral antibiotics used to treat mild to moderate skin and soft tissue infections in adults in the emergency department setting without systemic signs of infection (eg, fever, tachycardia, tachypnea, increased white blood cells). Mild purulent infections without systemic signs of infection may be treated with incision and drainage without antibiotics. MSSA indicates methicillin-sensitive *Staphylococcus aureus*; MRSA, methicillin-resistant *Staphylococcus aureus*; penicillin VK, penicillin V potassium; TMP/SMX, trimethoprim/sulfamethoxazole. Adapted, with permission, from LaPlante K. Providence Veterans Affairs Medical Center Antimicrobial Guide Empiric Therapy, 5th edition; and reference 22.

The AP reviewed existing guidelines, including the 2014 IDSA SSTI guidelines as well as recent published literature, and concurred that there is a need to revisit the 2014 IDSA guidance for the treatment of SSTIs specifically for the ED setting, particularly given the evolution of the epidemiology of SSTIs and treatment options over the decade since the IDSA guidelines were last updated. The AP focused their review on the selection of oral antibiotics but recognized that long-acting, intravenous antibiotics approved for use in the community setting may be appropriate in some situations. An updated list of oral agents for use in adults to treat SSTIs is included in Figure 1.

The 2014 IDSA SSTI guidelines recommend characterizing SSTIs as either nonpurulent or purulent, and the AP concurred with this initial risk stratification of patients with SSTI in the ED.^1^ The AP felt this approach is particularly important in the ED setting, where treatment is often empiric, and therefore agents should be selected to target suspected pathogens. Cellulitis and erysipelas are the most common nonpurulent SSTIs and are caused in most cases by streptococci. In contrast, purulent SSTI are most often caused by *S. aureus,* and the presence of MRSA is often of clinical concern.^9^ Though less prevalent than gram-positive organisms, gram-negative organisms may also be implicated in at-risk patients (eg, patients with diabetes, chronic kidney disease, or heart failure),^5^ with guidelines recommending use of an agent expected to have activity against suspected pathogens (eg, *Escherichia coli)*.

For patients who are not being admitted but, rather, are being discharged for outpatient therapy, the AP unanimously agreed oral antibiotics are preferred. Certain oral antibiotics listed in the 2014 IDSA SSTI guidelines are “strongly” recommended for use based on the GRADE (Grading of Recommendations Assessment, Development and Evaluation) system, indicating that they can be an option for most patients in most circumstances.^5^ Guideline-concordant outpatient oral antibiotics for nonpurulent SSTIs include penicillin V potassium, cephalexin, dicloxacillin, and clindamycin. Oral agents recommended by the 2014 IDSA SSTI guidelines for purulent SSTIs when MRSA is a concern include trimethoprim/sulfamethoxazole, clindamycin, linezolid, and tetracyclines (ie, doxycycline, minocycline). Since the 2014 IDSA SSTI guidelines, new agents with MRSA activity that have been approved include dalbavancin, oritavancin, delafloxacin, tedizolid, and omadacycline. Although there are many oral antibiotics in our armamentarium for the treatment of patients with SSTIs, there are important patient-specific and drug-specific effectiveness and safety considerations with many of the oral drugs, including newer agents, particularly when treating purulent SSTIs (Tables 1 and 2). However, there is limited real-world evidence currently to fully substantiate these safety and tolerability differences. Finally, some agents, particularly branded products, may pose cost and accessibility issues (eg, require prior authorization or access through a limited distribution network) relative to other agents.

**Table 1.** Examples of Patient-Specific and Local Considerations for Use of Oral Antibiotics for Treatment of SSTIs in Emergency Department Setting

Key consideration	Examples of patient-specific considerations
Empiric coverage for common, resistant pathogens	Has prior antibiotic therapy failed? Is there a high level of community resistance to clindamycin in S*taphylococcus aureus*? Is there a high level of community resistance to tetracycline for beta-hemolytic streptococci?
Patient comorbidities or age ≥65 years	Does the patient have renal dysfunction, or is the patient at high risk for renal dysfunction (due to, eg, age ≥65 years or diabetes)? Is the patient unable to tolerate alternative therapy (due to, eg, allergy history, high serum potassium level with TMP/SMX use)? Is an agent associated with a lower risk of CDI available, especially in older patients or those with a history of CDI?
Potential for drug interactions	Is the patient taking a serotonergic drug that may interact with linezolid?
Improved adherence	Would the patient benefit from improved adherence with a once-daily dosing regimen?
Accessibility	Are there drug cost or other accessibility issues (eg, required prior authorization or access through a limited distribution network)?

Abbreviations: CDI, *Clostridioides difficile* infection; SSTI, skin and soft tissue infection; TMP/SMX, trimethoprim/sulfamethoxazole.

**Table 2.** Examples of Drug-Specific Considerations for Use of Oral Antibiotics for Treatment of SSTIs in Emergency Department Setting

Drug(s)	Examples of drug-specific considerations
TMP/SMX	Generally, TMP/SMX is associated with high in vitro susceptibility rates among *Staphylococcus aureus* isolates, but some experts question its clinical role in SSTIs based on high treatment failure rates and supportive mechanistic concerns^10^ Sulfonamide allergy Comorbidities (eg, renal impairment)
Clindamycin	High nonsusceptibility rates among MRSA isolates (nearly 60% in some regions)^11^ Gastrointestinal effects including *Clostridioides difficile* superinfection
Tetracyclines (minocycline, doxycycline, omadacycline)	Gastrointestinal intolerance
Fluoroquinolones (delafloxacin)	Consider in cases of water exposure to cover *Pseudomonas aeruginosa* and related gram-negative rods Warnings include tendinitis and tendon rupture, peripheral neuropathy, and central nervous system effects^12^ Fluoroquinolone class as a whole carries a formidable list of warnings from FDA^13^
Oxazolidinones (linezolid, tedizolid)	Linezolid has safety and tolerability concerns when used in both the short term (eg, hypoglycemia) and longer term (eg, thrombocytopenia, neuropathy)^14,15^ Potentially clinically relevant drug-drug interactions between linezolid and commonly prescribed serotonergic agents resulting in serotonin syndrome^14^ Compared to linezolid, tedizolid appears to be associated with a lower incidence of adverse effects including hematologic toxicities, as well as substantially lower potential for drug interactions with serotonergic agents^16^

Abbreviations: FDA, US Food and Drug Administration; SSTI, skin and soft tissue infection; TMP/SMX, trimethoprim/sulfamethoxazole; MRSA, methicillin-resistant *Staphylococcus aureus*.

The AP also noted that there are unique pressures in the ED setting that influence treatment decisions. For example, efficient throughput is a major priority in the ED, and it is critical that appropriate patients are expeditiously discharged to home on optimal and cost-effective therapy.^17^ The AP believes there needs to be a greater focus on care of patients with mild and moderate SSTIs, who are primarily discharged to home, given the opportunity for enhanced antibiotic stewardship efforts in this patient population. Organization-specific treatment algorithms reflecting expert opinion and incorporating available oral antibiotics, including those approved in the last decade, should be developed and should include considerations for severity of illness, local antibiotic susceptibility patterns, patient comorbidities, cost, and accessibility.

## The opportunities and challenges of antibiotic stewardship in the ED

The AP agreed that the creation or updating of institution-specific guidelines and their implementation can be accomplished through an effective ED pharmacist–led antibiotic stewardship program.^18,19^ The knowledge and expertise of the stewardship team are essential to ensuring their implementation. Practical considerations are required for successful implementation and should include the capabilities and quality priorities of the care setting, knowledge of local susceptibility patterns, and patient-specific considerations (Table 2). Developing treatment guidelines that identify reasonable care approaches and monitoring the performance of these guidelines over time creates opportunities for improvement in prescribing practices and trends at the local level. One core element of an effective stewardship program in the ED is the ability to track and report prescribing practices and provide regular feedback to clinicians to optimize them.^20,21^ This audit and feedback process can be accomplished by conducting a meaningful medication-use evaluation that considers the real-world application of evidence specific to the setting and informs implementable recommendations for improvement. Process measures specific to the ED setting can include throughput times; readmission rates; reduction in treatment failures (eg, recurrences, reinfections, or hospital admissions); occurrence of, or reduction in, antibiotic-associated adverse events (eg, *Clostridioides difficile*); duration of therapy; increased medication adherence; and improved patient satisfaction.^22^

The AP recognized there are unique challenges related to the care of patients with SSTIs in the ED relative to other healthcare settings. For example, when selecting antibiotic therapy, there is a need to assess patient insurance coverage and prior authorization requirements. It may also be challenging to follow up with the patient or to assess outcomes once they are discharged, and there is often a lack of interoperability and integration of required data. The AP suggested strategies to address the unique antibiotic stewardship challenges in the ED setting, including:

- Aligning the organization’s goals with process measures
- Aligning formularies with payers and community pharmacies
- Communicating the treatment plan with primary care providers
- Working to enhance interoperability and exchange of information across settings and providers, including point-of-care prescription coverage and cost information

Convening a local institution-specific multidisciplinary group of stakeholders to reach a consensus on treatment guidelines is highly recommended to achieve the best possible outcomes for an organization, its antibiotic stewardship program, and, most importantly, its patients.^21^ The understanding of baseline antibiotic prescribing patterns and practices in SSTI gained by conducting a medication-use evaluation at an institution is critical to monitoring the implementation of institution-specific guidelines and reviewing adherence to those guidelines. The following questions are recommended when conducting any antibiotic-use review in the ED setting and can be adapted specifically to SSTI:

- Do institution-specific guidelines exist?
- Do institution-specific guidelines reflect current evidence?
- Is prescribing consistent with institution-specific guidelines?
- Do the guidelines (and relevant drug formulary options) align with the broader goals of the ED (eg, increase throughput and decrease repeat visits or readmissions)?
  - Are there any organizational performance goals and quality metrics for the ED (eg, time to triage, time to evaluation by a provider, time to disposition) relevant to antibiotic stewardship for SSTI?
- Do empiric treatment recommendations minimize the risks of clinical failure and/or recurrences?
  ◦ Do institution-specific guidelines reflect institution and community resistance patterns?
- Do preferred antibiotic treatments align with the hospital and outpatient formularies?
- If prior authorization is required, how is this coordinated with payors and pharmacies?
- Are there ways to assess patient outcome differences based on protocol adherence?
- Are patient-specific considerations (eg, concomitant diseases, potential drug interactions, adverse events, ability to adhere to therapy) accounted for and considered to achieve the best opportunity for adherence and meeting treatment goals?
  ◦ Is intravenous therapy an outpatient option (eg, is there access to an outpatient infusion center)?
  ◦ Is oral therapy and treatment at home an option?
  ◦ Is once-daily dosing likely to increase patient adherence?
  ◦ Are there concerns of renal or hepatic insufficiency (eg, diabetes, age ≥65 years)?
  ◦ Are there any notable drug-drug interactions with other medications prescribed? Is the interaction significant?
  ◦ Can the patient access the medication prescribed (eg, concerns related to medication cost)?

## Conclusion

SSTI treatment considerations in the ED supported by evidence-based guidelines, including guidance on optimal oral antibiotic selection, can be a useful tool to improve the quality and efficiency of care, enhance patient-centric outcomes and satisfaction, decrease healthcare costs, and reduce overuse of antibiotics. While national guidelines serve as the gold standard on which to base these treatment decisions, local institutional guidelines must be regularly updated and should be based on local antibiotic resistance patterns, local drug formulary plans, and patient-specific clinical and logistical considerations. Convening a team of experts locally to establish institution-specific guidelines as part of a comprehensive antibiotic stewardship program can ensure patients receive the most appropriate therapy for the treatment of SSTI. Furthermore, reviewing antibiotic utilization in the treatment of SSTIs in this setting within the context of institutional guidelines can ensure optimal treatment for patients.

## Data Availability

No new data were generated or analyzed in support of this article.

## References

[1] Kaye KS , Petty LA, Shorr AF, Zilberberg MD. Current epidemiology, etiology, and burden of acute skin infections in the United States. Clin Infect Dis. 2019;68(suppl 3):S193-S199. doi:10.1093/cid/ciz002 30957165 PMC6452002

[2] Weiss AJ , Jiang HJ. Statistical brief #286: most frequent reasons for emergency department visits. Agency for Healthcare Research and Quality. Published December 2021. Accessed July 6, 2023. https://hcup-us.ahrq.gov/reports/statbriefs/sb286-ED-Frequent-Conditions-2018.jsp

[3] Golan Y. Current treatment options for acute skin and skin-structure infections. Clin Infect Dis. 2019;68(suppl 3):S206-S212. doi:10.1093/cid/ciz004 30957166 PMC6451992

[4] US Food and Drug Administration. Guidance for industry: acute bacterial skin and skin structure infections: developing drugs for treatment. Published October 2013. Accessed March 21, 2024. https://www.fda.gov/media/71052/download

[5] Stevens DL , Bisno AL, Chambers HF, et al; Infectious Diseases Society of America. Practice guidelines for the diagnosis and management of skin and soft tissue infections: 2014 update by the Infectious Diseases Society of America. Clin Infect Dis. 2014;59(2):e10-e52. doi:10.1093/cid/ciu444. Published correction in Stevens DL, et al. *Clin Infect Dis*. 2015;60(9):1448. 24973422

[6] Centers for Disease Control and Prevention. Active Bacterial Core surveillance (ABCs). Accessed July 16, 2023. https://www.cdc.gov/abcs/index.html

[7] Sullivan T , de Barra E. Diagnosis and management of cellulitis. Clin Med (Lond). 2018;18(2):160-163. doi:10.7861/clinmedicine.18-2-160 29626022 PMC6303460

[8] Sakoulas G. Expert update on acute bacterial skin and skin structure infection treatment options in the community setting. J Fam Pract. 2022;71(suppl 1 bacterial):S2-S8. doi:10.12788/jfp.0344 35413233

[9] Singer AJ , Talan DA. Management of skin abscesses in the era of methicillin-resistant *Staphylococcus aureus*. N Engl J Med. 2014;370(11):1039-1047. doi:10.1056/NEJMra1212788 24620867

[10] Proctor RA. Role of folate antagonists in the treatment of methicillin-resistant *Staphylococcus aureus* infection. Clin Infect Dis. 2008;46(4):584-93. doi:10.1086/525536. PMID: 18197761 18197761

[11] Healthcare-Associated Infections–Community Interface, Centers for Disease Control and Prevention. Emerging Infections Program (EIP) Network report: invasive *Staphylococcus aureus*, 2016. Updated February 13, 2020. Accessed September 15, 2023. https://www.cdc.gov/hai/eip/pdf/2016-MRSA-Report-508.pdf

[12] Baxdela. Prescribing information. Melinta Therapeutics, LLC; 2021. Accessed October 8, 2023. https://baxdela.com/docs/baxdela-prescribing-information.pdf

[13] US Food and Drug Information. Fluoroquinolone antimicrobial drugs information. Accessed March 20, 2024. https://www.fda.gov/drugs/information-drug-class/fluoroquinolone-antimicrobial-drugs-information

[14] Zyvox. Package insert. Pfizer Inc.; 2023.

[15] Viswanathan P , Iarikov D, Wassel R, Davidson A, Nambiar S. Hypoglycemia in patients treated with linezolid. Clin Infect Dis. 2014;59(8):e93-e95. doi:10.1093/cid/ciu487 24965346

[16] Wong E , Rab S. Tedizolid phosphate (sivextro): a second-generation oxazolidinone to treat acute bacterial skin and skin structure infections. P T. 2014;39(8):555-579. 25136252 PMC4123804

[17] Hoot NR , Aronsky D. Systematic review of emergency department crowding: causes, effects, and solutions. Ann Emerg Med. 2008;52(2):126-136. doi:10.1016/j.annemergmed.2008.03.014 18433933 PMC7340358

[18] Choi PW , Benzer JA, Coon J, Egwuatu NE, Dumkow LE. Impact of pharmacist-led selective audit and feedback on outpatient antibiotic prescribing for UTIs and SSTIs. Am J Health-Syst Pharm. 2021;78(suppl 2):S62-S69. doi:10.1093/ajhp/zxab110 33769435

[19] Gibbons JA , Smith HL, Kumar SC, et al. Antibiotic stewardship in the treatment of skin and soft tissue infections. Am J Infect Control. 2017;45(11):1203-1207. doi:10.1016/j.ajic.2017.05.013 28732743

[20] Fay LN , Wolf LM, Brandt KL, et al. Pharmacist-led antimicrobial stewardship program in an urgent care setting. Am J Health-Syst Pharm. 2019;76(3):175-181. doi:10.1093/ajhp/zxy023 30689745 PMC6366123

[21] Sanchez GV , Fleming-Dutra KE, Roberts RM, Hicks LA. Core elements of outpatient antibiotic stewardship. MMWR Recomm Rep. 2016;65(RR-6):1-12. 10.15585/mmwr.rr6506a1 27832047

[22] ASHP Research and Education Foundation. ASHP Medication-Use Resource guide: treatment of skin and soft tissue infections in the emergency department: evaluating omadacycline use. Accessed July 23, 2023. https://www.ashpfoundation.org/-/media/REF/Research/PDFs/MUE_Resource-Guide-Omadacycline.pdf

